# P-1619. The Impact of mask mandate during COVID-19 pandemic on pediatric hospitalization for acute respiratory illnesses in a community hospital

**DOI:** 10.1093/ofid/ofaf695.1796

**Published:** 2026-01-11

**Authors:** Shreya Sodhani, Kedar Tilak, Erica Ray, Louisdon Pierre, Adebayo Adeyinka, Noah Kondamudi

**Affiliations:** University of Iowa, Iowa CIty, Iowa; Children's Mercy Hospital, Overland Park, KS; The Brooklyn Hospital Center, New York, New York; The Brooklyn Hospital Center, New York, New York; The Brooklyn Hospital Center, New York, New York; The Brooklyn Hospital Center, New York, New York

## Abstract

**Background:**

During the COVID-19 pandemic, public health measures were implemented to prevent the transmission of the virus. One such measure was the mask mandate which was introduced in New York City in April 2020. Use of masks by parents and family members as well as by children themselves helps prevent the transmission of respiratory illnesses that spread through droplets. The principal objective of this study is to assess whether the implementation of a mask mandate during the COVID-19 pandemic had an impact on the number of pediatric hospitalizations for acute respiratory illnesses.

**Figure:**
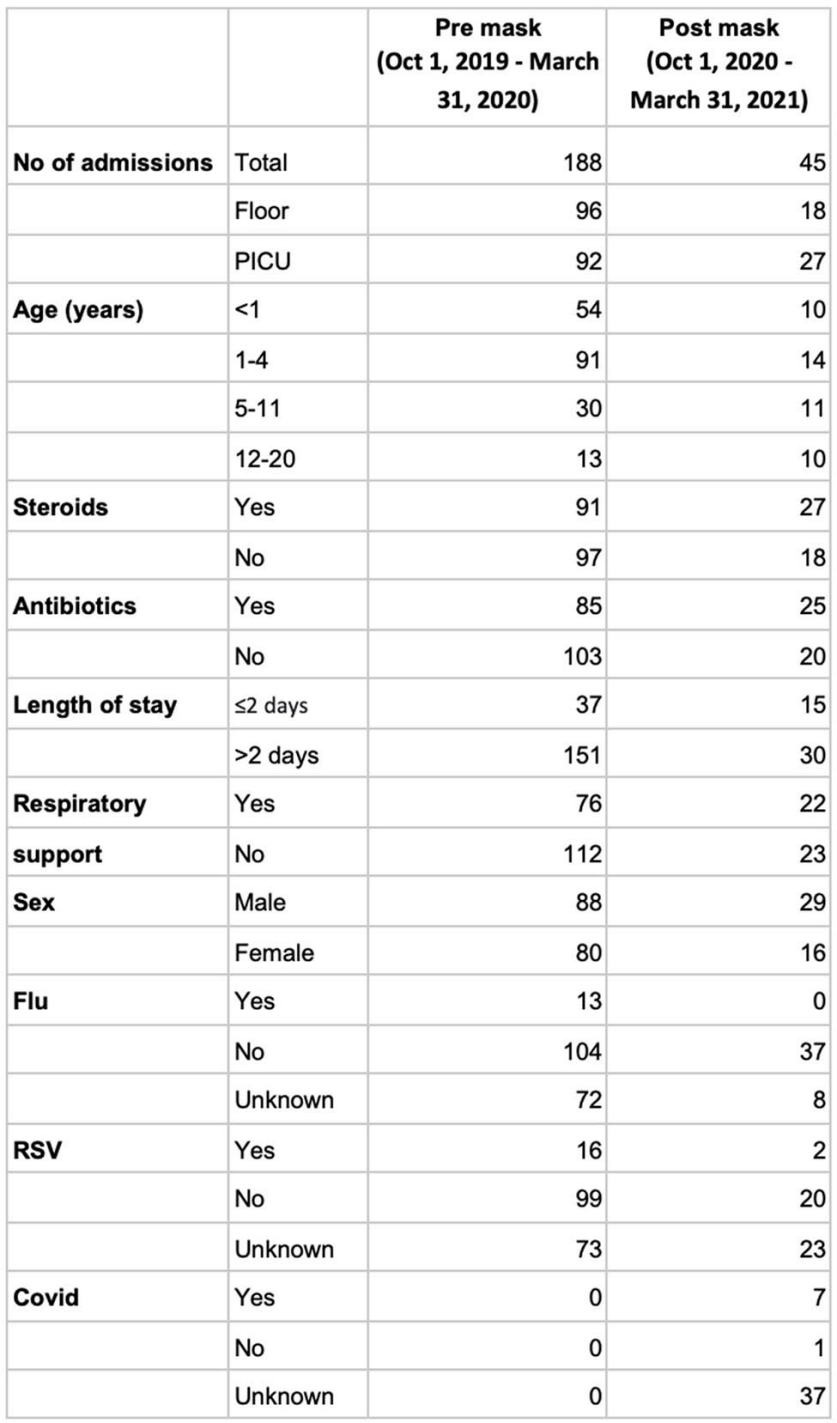
Pre-mask vs Post-mask Comparison of Variables

**Figure:**
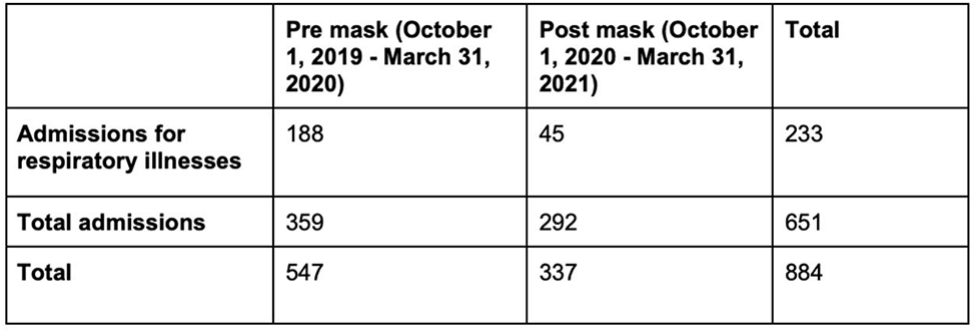
Patient Demograpghics Pre-mask vs Post-mask Admissions The p-value is < 0.00001. The result is significant at p < 0.05

**Methods:**

This study is a retrospective analysis of the electronic medical record (EMR) of all children from birth to twenty years admitted to the pediatric floor or the pediatric intensive care unit (PICU) for acute respiratory illnesses including COVID-19, to our community hospital between October 1, 2019 - March 31, 2020 (pre-pandemic) and October 1, 2020 and March 31, 2021 (after mask mandate during the pandemic). Data was analyzed to explore if there are any associations in the two groups related to age, sex, length of hospital stay, severity of illness based on need for respiratory support, use of antibiotics and steroids. SPSS version 25 was used for statistical analysis.

**Figure:**
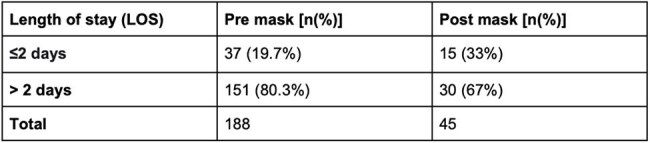
Pre-mask vs Post-mask Length of Stay The p-value is 0.048. The result is significant at p <0 .05

**Results:**

We observed a sharp decline in pediatric admissions for respiratory illnesses from 188 before the mask mandate to 45 after the implementation of the mask mandate (Table 2; p < 0.05). We also noted a decrease in the length of stay, where 80.3% of patients had a hospital stay of more than 2 days before the mask mandate compared to 67% after the mask mandate (Table 3; using chi square test; p< 0.05). Demographics and other variables including respiratory support, antibiotics and steroid use are as shown in Table 1.

**Conclusion:**

After the implementation of the mask mandate during the COVID-19 pandemic, there was a significant decline in pediatric hospitalizations for acute respiratory illnesses. This suggests that wearing masks can be an effective way to prevent the transmission of respiratory illnesses, including COVID-19. This study also highlights the importance of adherence to public health measures to reduce healthcare burden especially during a crisis like the COVID-19 pandemic.

**Disclosures:**

All Authors: No reported disclosures

